# Hypoglycemic effect of polysaccharides with different molecular weight of *Pseudostellaria heterophylla*

**DOI:** 10.1186/1472-6882-13-267

**Published:** 2013-10-16

**Authors:** Juan Hu, Wensheng Pang, Jinlong chen, Shaowei Bai, Zhenzhu Zheng, Xiaohua Wu

**Affiliations:** 1 The Institute of Drug Research, Fujian Academy of Traditional Chinese Medicine, 282 Wusi north Road, Fuzhou, Fujian Province 350003, People’s Republic of China; 2 The College of Pharmacy, FuJian University of Traditional Chinese Medicine, Fuzhou 350122, People’s Republic of China; 3 The Second People’s Hospital of Fujian Province, Fuzhou 350003, People’s Republic of China; 4 Fujian Lijiexun Pharmaceutical Co., Ltd., Zherong County 355300, People’s Republic of China

**Keywords:** *Pseudostellaria heterophylla*, Polysaccharides, Molecular weight range, Anti-diabetes, Biomarkers

## Abstract

**Abstracts:**

**Background**

The aims of this study were to evaluate the antidiabetic activity and to detect molecular size of *Pseudostellaria heterophylla* polysaccharide (PHP). *Pseudostellaria heterophylla* is a medicine extensively used in traditional Chinese medicine formulas to treat diabetes and its complications.

**Methods**

Molecular weight of PHP was determined by gel permeation chromatography combined with phenol-sulphuric acid method and the monosaccharides composition was determined by HPLC with a precolumn derivatization. Four polysaccharides with different molecular weight were compared for hypoglycemic active on two animal models both high does alloxan induced type1 diabetic mellitus (T1DM) and high-fat/lower does streptozotocin induced type2 diabetic mellitus (T2DM). Blood sugar, glucose tolerance, and insulin tolerance were detected. Rat serum IL-1β, IL-2, IL-10, Leptin, TNF-α, Acrp30 and CRP were also analyzed by sandwich-ELISA approaches to preliminary probe the hypoglycemic mechanism of PHP.

**Results**

The hypoglycemic effects related to molecular size of polysaccharide were more effective against T2DM than T1DM. PHP comprise four monosaccharides of galacturonic acid, glucose, galactose and arabinos. T2DM rats daily receiving oral dose of polysaccharide(100 ~ 400 mg/kg) with 50 ~ 210 kDa molecular weight (PF40) could not only significantly lower blood sugar but also reduce total triglyceride level in serum. PF40 improves in insulin tolerance inhibited the expression of some biomarkers including inflammatory cytokine TNF-α and elevated anti-inflammatory cytokine IL-10, regulated adiponectin Acrp30 and leptin.

**Conclusions**

PF40 prevent the cascade of inflammatory events in the treatment of T2DM to block overweight progresses to obesity.

## Background

Diabetes mellitus (DM) is a serious chronic metabolic disease and be divided into two major types by etiology, namely type1 diabetes mellitus (T1DM) or type2 diabetes mellitus (T2DM). It is a global disease that a thorny problem in the world medicine. Increasing trend of greater number and/or younger DM patients is for seen due to greater prevalence of obesity and sedentary life style [1]. Diabetes needs long-term treatments in order to have their conditions brought under control and to prevent complications. Exacerbate symptoms associated with hyperglycemia is primarily attributed to microvascular and macrovascular changes which can cause atherosclerosis, retinopathy, renal failure, and peripheral artery diseases. Once complications are allowed to creep in, the outcome is in danger [2]. Commonly practiced pharmacologic treatment of DM includes take hypoglycemic agents and insulin. But to date, insulin cannot be used orally and its repeated injections have many undesirable adverse effects. There are many oral hypoglycemic agents for the treatment of diabetes, such as biguanides and sulfonylureas; biochemical drugs,such as insulin-secretagogues,insulin sensitivity improvement factor, insulin-like growth factor,aldose reductase inhibitor, α-glucosidase inhibitors and protein glycation inhibitor. However, intake of these drugs also causes side effects, for example, hypoglycemia, lactic acid intoxication, gastrointestinal upset, and are not effective in lowering the blood sugar in chronic diabetic patients [3]. There is an increasing demand by patients for the use of plant medicinal, folk medicine, traditional medicine of ethnic minorities, as well as other dietary modulators to help control the symptoms of diabetes [4-8].

In Chinese clinical medicine, “the diabetes mellitus” belongs to the “wasting thirst sickness category”, which is known as Xiaokezheng with respect to the sign of imbalance of Yin, Yang and Qi. Patients usually suffer from dryness of the mouth and increased thirst, blood stasis and constipation [9]. The medicinal information on traditional includes about 400 Chinese herbs, a number of Chinese formulas, and Chinese patent medicines used for controlling DM and its complications based on the theories to tendency in eliminating heat nourishing yin, then supplementing Qi and nourishing Yin [10,11].

Polysaccharides are the most abundant and the most diverse materials found on earth. Drugs and health foods made of polysaccharides have been a research hotspot in the field of life sciences. Polysaccharides feature prominently anti-diabetes in many Chinese herbs. Radix astragali, radix pseudostellariae are considered of utmost importance two herbs in traditional medicine to treat at DM. It had been made a deep study that the structures and their mechanism of radix astragali of polysaccharides anti-diabetes [12]. *Acanthopanax senticosus* polysaccharide oral administration had an efficacious amelioration effect on the antioxidant status in alloxan-induced diabetic mice. The polysaccharide from *Salvia miltiorrhiza* Bunge can protect against the development of T2DM and improve insulin resistance via reduction of oxidative stress. The polysaccharide from *Ophiopogon japonicus* possess potent antioxidant activity and can protect the liver and kidneys from the injurious effects of diabetes [13,14]. These were believed, through pharmacological studies, that herb polysaccharides restore the functions of pancreatic tissues causing an increase in insulin output by the functional beta cells (β-cell), thus lower the blood glucose levels. These polysaccharides have also been shown to improve the sensitivity of peripheral cells to circulating insulin.

*Pseudostellaria heterophylla* (Miq.) Pax, as described in the Pharmacopoeia of People’s Republic of China, Strengthens Qi and generates Fluids. Use for lack of appetite from deficient spleen; fatigue, palpitation, profuse sweating; thirst from lung Yin deficiency [15]. In the previous studies, the major components of *Pseudostellaria heterophylla* include volatile compounds, saponins, polysaccharides, cycle peptides, amino acids, minerals, etc., and these chemical compounds are related to diverse biological activities, such as suppressing cough, immunologic enhancement and so on [16], it is widely used as anti-diabetes drugs in Chinese clinical. *Pseudostellariae heterophylla* is rich in polysaccharides. The crude polysaccharide of *Pseudostellaria heterophylla* (PHP) effects on glucose and lipid metabolism [17-19]. In the previous studies, the researches in most cases pitched at a simple level, but the benefits of R&D of PHP are potentially huge.

The feasibility of utilizing inflammatory markers in screening T2DM risk can be substantiated from numerous experimental, clinical and epidemiological observations demonstrating that the ability of inflammatory factors to predict the disease independently from established risk factors. Overweight sets the stage for low-grade chronic inflammation, with adiponectin levels decreasing while resistin, FFAs and TNF-α increase. As overweight progresses to obesity, continued inflammation further leads to elevated CRP, fibrinogen, IL-6, IL-1β and haptoglobin. Obesity can be complicated by metabolic dysregulation (metabolic syndrome) to develop frank T2DM where LDL-cholesterol and triglyceride (TG) levels increase, HDL-cholesterol levels deceases and hypertension and IGT manifest. Throughout the pathologic continuum from overweight to T2DM, insulin resistance increases progressively. T2DM is linked to fourfold higher risk. Public health initiatives aimed at preventing and controlling T2DM should be targeted towards the early stages of the disease, to prevent obesity and the cascade of inflammatory events that eventually leads to the clinical manifestation of T2DM [20].

It had been reported that PHP could decrease levels of blood glucose on diabetic rats. Polysaccharide of different molecular weight whether has same lower blood glucose effects; this incited us to perform further studies on anti-diabetic actions of the molecular weight size of polysaccharides. In this paper, crude polysaccharide was degraded to get three low molecular weight fractions and fed to type 1 diabetics mice induced by alloxan and type 2 diabetics obese rats, on sweet, high-fat diet-induced by low dose streptozotocin for 30 days. We accurately screen more active hypoglycemic faction and detect lipid profiles and the biomarkers such as pro-inflammatory cytokines and chemokines to prospect novel strategies for polysaccharides prevention diabetes mellitus.

## Methods

### Materials and reagents

*Pseudostellaria heterophylla* was provided from GAP bases of Fujian Lijiexun Pharmaceutical Co., Ltd., Zherong County, Fujian Province of China in 2010. The plant was identified by Dr. M. Jin and a voucher specimen (No. 2010131037S) is deposited at the Fujian Provincial Institute for Drug Control, Fuzhou City, Fujian Province, China. The plant material was dried in a ventilated oven at 40°C for 48 h. Dextran G100/Sepha and the various standards with different molecular weight (blue dextran, T10, T40, T70, and T500) were purchased from Pharmacia (NJ, USA). Bovine serum albumin, alloxan and streptozocin were purchased from Sigma (MO, USA). Phenol, *n*-butyl alcohol, chloroform, Coomassie brilliant blue G-250 and sulfuric acids were purchased from Sinopharm Chemical Reagent Co. (Shanghai, China). All reagents and solvents were of analytical reagent grade and used without further purification unless otherwise noted. All aqueous solutions were prepared using newly double-distilled water.

### Preparation of crude polysaccharide and ethanol classification

The polysaccharide was extracted by water and alcohol precipitation method. The dried medicinal materials was pulverized and passed through a 40-mesh sieve. The sample powder was immersed in 85% ethanol for 1 h (1:8, *m/v*) and extracted under reflux for three time (every 2 h). Dregs were extracted with water (1:8, *m*/*v*) for three times (every 2 h). Then the extractions were filtered and the combined filtrate was centrifuged at 4000 rpm for 10 min to remove the residues. The supernatant was concentrated to 1/20 volume in a rotary evaporator under reduced pressure and precipitated with 90% ethanol. The precipitation was collected by centrifugation and dissolved in warm water. The crude polysaccharide fluid was deproteinized by Sevage method with *n*-butyl alcohol: chloroform equal 25:5:1(*v*/*v*) shaken the container for 5 min, placed to stratification and then was centrifuged at 4000 rpm for 5 min. The supernatant was concentrated to 1/10 volume in a rotary evaporator under reduced pressure and precipitated with 90% ethanol, put it in a resting position for 24 h and centrifuged at 4000 rpm for 5 min. The precipitation was freeze-dried to yield total polysaccharide powder from *Pseudostellaria heterophylla*.

Total polysaccharide, that was crude polysaccharide, abbreviated to PH-TP. PH-TP was dissolved in warm water, were subjected to a sequential precipitation with ethanol at 40%, 60% and 90% (the supernatant after precipitated by 60 percent of ethanol) to obtain three fractions of FP40, FP60 and FP90 (the small molecules polysaccharide). Two or more polysaccharides are expressed in PHPs.

### Analysis of components in polysaccharide

At 110°C temperature for 6 h, polysaccharide was hydrolyzed by 2 mol/mL trichloroacetic acid. High performance liquid chromatography (HPLC) method with pre-column derivatization was established for the determination of the monosaccharides of PHP from the hydrolyzate and their weight percentage content.

### Determination of the polysaccharide content and molecular weight

Polysaccharide was deproteinized by repeated freeze-thaw method. The total sugars were determined by the phenol-sulphuric acid method with d-glucose as standard. The soluble protein was determined by the Coomassie brilliant blue G-250 method with bovine serum albumin as a standard. Sephadex G-100 gel filtration chromatography column (1 cm × 50 cm, 1 cm × 80 cm), a Model DBS-160 automatic collector, and Model TBP-50A constant flow pump were used. The mobile phase was 0.5 mol/L NaCl with flow rate of 0.3 ml/min, which dextrans (blue dextran, T10, T40, T70, and T500) were used as references, drew the standard curve from. The average molecular weights (MW) of PHPs were determined.

### Production of diabetes model and experimental design

Wistar rats and KM mice were used for the animal model experiment, purchased from Shanghai SLAC Laboratory Animal Co., Ltd. (License number: SCXK (Shanghai) 2009–0005). Animals were housed in an environmentally-controlled room at a temperature of 22 ± 1°C, relative humidity 65-70% with feed and water ad libitum. The animal studies were approved by the Fujian Institute of Traditional Chinese Medicine Animal Ethics Committee, Fuzhou, China (No. FJZYY-AEEI 2010012). The experimental procedures were carried out in accordance with the Guidelines for Animal Experimentation of Fujian University of Traditional Chinese Medicine (Fuzhou, China).

#### Type 1 diabetic model

Male KM mice, weighing 18–22 g, were injected with ALX 95 mg/kg b.w. injection in caudal vein route. Blood sugar levels were estimated after 3 days to establish type 1 diabetic model; this also was non obese diabetes (NOD) [21]. Mice having sugar level>20 mmol/L were selected for experiments.

#### Type 2 diabetic model

Adult male Wistar rats were fed with high-fat and high-sugar diet (Feed formula: by 10% lard, 2.5% cholesterol, 1% sodium cholate, 20% sucrose, and 66.5% of the basic feed). Rats were injected with STZ in citrate buffer (pH 4.5) at a dose below 50 mg/kg b.w. in abdomen route after 4 weeks to establish type 2 diabetic model; this also was high fat diabetes (HFD) [21]. Rats having sugar level>11.1 mmol/L were selected for experiments.

#### Experimental groups

Fourteen groups of mice/rats, ten in each received the following treatment schedule. Mice treated with polysaccharide which was administrated at high dose of 4 g/kg (HD), moderate dose of 2 g/kg (MD), and low dose of 1 g/kg (LD). Rats treated with polysaccharide which was administrated at high doses of 3 g/kg, moderate dose of 1.5 g/kg, and low dose of 0.5 g/kg.

Group I: (Control) normal mice or rats.

Group II: (Diab.) ALX or STZ induced diabetic model.

Group III: (Positive) animals were given metformin 0.23 g/kg (mice), 0.15 g/kg (rats).

Group IV: (PH-TP) diabetic animals treated with PH-TP, HD, MD, and LD.

Group V: (PF40) diabetic animals treated with PF40-HD, PF40-MD, and PF40-LD.

Group VI: (PF60) diabetic animals treated with PF60-HD, PF60-MD, and PF60-LD.

Group VII: (PF90) diabetic animals treated with PF90-HD, PF90-MD, and PF90-LD.

### Assay of glucose level and lipid profiles

The blood glucose, total cholesterol (TC), triglyceride (TG), low density lipoprotein (LDL), and high density lipoprotein (HDL) were determined, respectively. All the manipulations followed the directions of the commercially available kits (Jiancheng Institute of Biotechnology, Nanjing, China). The operation according to specification and results were expressed in mmol/L.

### Oral glucose tolerance and insulin tolerance test

The glucose tolerance test (OGTT) evaluates the ability to respond appropriately to a glucose challenge. OGTT was conducted in control and treated rats, after overnight fast, blood samples were collected from the rats’ tail vein (control and treated groups) to obtain baseline blood glucose levels. Subsequently, all the rats in groups were fed with glucose (2 g/kg b.w.). Blood samples were taken by distal venesection of the tail vein at interval of 30 min up to 2 h for glucose estimation.

Insulin tolerance test (ITT) evaluates insulin sensitivity. After overnight fast, blood samples were collected from the rats’ tail vein (control and treated groups) to obtain baseline blood glucose levels. Rats were injected with insulin at a dose of 1.0 u/kg b.w. in abdomen route, blood samples were taken by distal venesection of the tail vein at interval of 30 min up to 2 h for glucose estimation.

### Biomarker analysis associated with diabetes by multiplex sandwich-ELISA

Collect venous blood in a hard plastic. Allow blood to clot at room temperature for 1 hour. Centrifuge the specimen at 1,600 × g for 15 minutes then carefully pipette the clear serum (supernatant) to a clean specimen tube using a Pasteur pipette. Aliquot into pre-labeled plastic, screw-cap vials and store at -80°C, avoid freeze-thaw cycles.

This test includes 7 analytes, C-reactive protein (CRP), adiponectin (Acrp30), interleukin-1beta (IL-1β), interleukin-1beta (IL-10), Leptin, and tumor necrosis factor (TNF-α). Multiplex Protein Biomarker Testing Systems were used in this testing project. This is a multiplex sandwich-ELISA in a planar, plate-based array format, including SignatureLUS CCD Imaging and ProArray Analysis System. Arrays used in this test were Aushon Rat Custom Array Kit, catalog No. 85849 (Rat IL-1β, IL-2, IL-10, Leptin, TNF-α, Acrp30, CRP).

### Statistical analysis

Differences in measurement data were compared with the analysis of variance test using SPSS/11.5 software. P values of less than 0.05 were considered to indicate a significant difference between treatments. All values are expressed as mean ± SD.

## Results and discussion

### Components and molecular weight of polysaccharides

The content of total sugar and protein of four PHPs were 41.08-47.28% and 0.17-0.25%, respectively. There were no significant differences in total polysaccharide content and protein contents of samples precipitated by different concentrations of alcohol. However, a notable difference was observed in the range of molecular weight of polysaccharide. Protein had been removed nearly; total sugar content was superior to protein content, indicating that the extractions by this method could be recognized as polysaccharides. The molecular weight distribution of polysaccharide ranged from 3.0 kDa to 212 kDa, the polysaccharides in 4 samples were determined, as shown in Table 1. Further research on hypoglycemic constituent in crude polysaccharide is by this paper.

**Table 1:** Some information of PHPs used in this work

**PHPs**	**Contents of protein (%)**	**Contents of total sugar (%)**	**Molecular weight (Da)**
PH-TP	0.25	44.40	7.0 × 10^3^ ~ 2.1 × 10^5^
PF40	0.19	47.28	5.2 × 10^4^ ~ 2.1 × 10^5^
PF60	0.21	41.08	1.0 × 10^4^ ~ 2.1 × 10^5^
PF90	0.17	45.89	3.0 × 10^3^ ~ 6.8 × 10^3^

**Figure 1:**
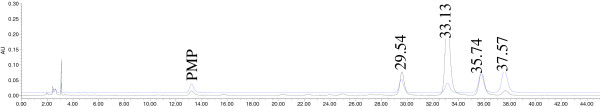
The chromatogram of 4 kinds of monosaccharides (blue-reference substance and black-PHF40 sample); the peaks from left to right were PMP, galacturonic acid, glucose, galactose and arabinos in the order of retention time.

According to the chromatographic peak of monosaccharides reference substance and samples, the composition of the monosaccharides in PHP was identified. HPLC analysis showed that polysaccharides of consists of galacturonic acid, glucose, galactose and arabinos; their weight percentage content was about 8.21%, 85.97%, 2.59%, 3.23%, respectively. Different molecular weight polysaccharides are composed of 4 monosaccharides, but different in content. The retention time (Rt) with each chromatographic peak was galacturonic acid 13.22, glucose 29.54, galactose 33.13 and arabinos 37.57 min; the Rt of PMP was about 13.22 min (Figure 1).

### Effect of polysaccharide on glucose homeostasis

#### Blood glucose level of type 1 diabetic mice

Blood glucose level was monitored using biochemistry analyzer on 0, 15th, and 21st day, blood samples were taken from retro orbital plexus. Blood glucose levels in type 1 diabetic mice were raised to 50 mmol/L as compared to normal control group mice on starting (0) day. After oral administration of PH-TP, PF40, PH60, and PH90 for 15 days, the elevated glucose levels remained static in four polysaccharide groups. 30^th^ day, at the end of treatment the value in the high dosage of polysaccharides, blood glucose level of diabetic mice moderately attenuate, were significantly differences compare with model control group (p<0.05). Compare with positive control group, but with little success (Table 2).

**Table 2:** Blood glucose level of type 1 diabetic mice and type 2 diabetic rats of treating with polysaccharides

**Groups**	**Doses**	**T1DM mice blood glucose levels(mmol/L)**			**Relative to model control (30**^**th **^**day)**	**T2DM rats blood glucose levels(mmol/L)**			**Relative to model control 30**^**th **^**day**
		**Day 0**	**Day 15**	**Day 30**		**Day 0**	**Day 15**	**Day 30**	
Normal control		7.7 ± 1.2	6.7 ± 1.6	5.4 ± 2.3		5.7 ± 2.1	5.60 ± 0.28**	3.91 ± 1.38**	
Model control		52.1 ± 7.6	51.3 ± 10.4	42.6 ± 2.8		23.9 ± 6.6	19.77 ± 3.69	25.10 ± 0.61	
Positive control		62.5 ± 9.8	24.4 ± 4.9	10.4 ± 6.0**	↓75.58%	23.5 ± 4.8	14.04 ± 2.55**	6.72 ± 1.57**	↓73.22%
PH-TP	H	54.6 ± 3.1	52.2 ± 1.6	37.9 ± 5.5*	↓11.03%	25.0 ± 2.4	18.69 ± 4.22c	14.84 ± 2.41**	↓40.87%
	M	56.0 ± 2.4	51.8 ± 6.8	48.8 ± 7.1^C^	↑14.55%	24.2 ± 2.5	16.73 ± 2.95c	16.67 ± 2.41**	↓33.59%
	L	56.8 ± 2.8	50.1 ± 5.2	40.1 ± 5.3^C^	↓5.87%	23.4 ± 5.8	16.68 ± 2.69c	12.95 ± 0.92**	↓48.41%
PF40	H	52.9 ± 5.5	50.8 ± 4.3	37.5 ± 8.7*	↓11.97%	24.0 ± 5.7	15.78 ± 5.05*	6.78 ± 5.47**	↓72.99%
	M	51.2 ± 4.1	49.4 ± 3.5	34.2 ± 8.0*	↓19.72%	25.2 ± 3.1	15.82 ± 3.67*	11.41 ± 2.89**	↓54.54%
	L	50.2 ± 6.3	47.6 ± 7.1	40.4 ± 7.3^C^	↓5.16%	23.3 ± 6.9	15.25 ± 1.95*	12.80 ± 1.15**	↓49.00%
PF60	H	52.8 ± 6.6	49.9 ± 2.7	37.4 ± 11.3*	↓12.21%	24.0 ± 4.4	19.82 ± 6.98c	13.73 ± 5.24**	↓45.30%
	M	54.9 ± 6.5	47.2 ± 4.1	40.1 ± 4.1^C^	↓5.87%	23.7 ± 5.1	16.05 ± 2.85*	14.98 ± 6.05**	↓40.32%
	L	51.7 ± 1.1	45.3 ± 5.9	41.5 ± 3.6^C^	↓2.58%	24.7 ± 1.1	12.99 ± 3.66*	12.82 ± 4.97**	↓48.92%
PF90	H	50.7 ± 1.3	50.5 ± 4.8	40.2 ± 14.3^C^	↓5.63%	23.9 ± 1.3	16.31 ± 2.24*	14.77 ± 3.73**	↓41.16%
	M	56.3 ± 2.0	52.4 ± 5.7	39.5 ± 5.0 ^C^	↓7.27%	25.3 ± 2.0	15.13 ± 2.53*	14.83 ± 2.29**	↓40.92%
	L	52.2 ± 0.99	47.6 ± 7.5	39.8 ± 5.2^C^	↓6.57%	22.2 ± 0.99	16.74 ± 2.33c	17.08 ± 3.56**	↓31.95%

Values are expressed as mean ± S.d., n = 10.

Significantly different from the model group at* P ≤ 0.05, ** P ≤ 0.01.

^C^versus model is NOT different.

The sign “↓” expresses fall and “↑” expresses rise.

#### Blood glucose level of type 2 diabetic rats

The raised levels of blood glucose declined sharply after oral administration of Metformin, PH-TP, PF40, PF60, and PF90. When comparisons were made between 0 day, and 30^th^ day of treated groups, there was highly statistically significant (P<0.01) decline in blood glucose levels, as shown in Table 2. If decline in blood glucose levels is to be the only indices, then treatment with PH-TP, PF40, PF60, PF90, and Metformin were highly effective in causing significant anti-hyperglycemic response in this strain of rats. As far as the relative efficacy is concerned, though all the four polysaccharides were extremely effective, PF40 has been proven the highest anti-hyperglycemic activity among all. There had been ever so little difference between the high dose of PF40 and Metformin, but small molecule polysaccharide (PF90) was less effective.

#### Serum lipids of rats treated with PF40

After treatment with the most effective PF40 at three doses of high, medial, and low i.g. for 30 days, serum insulin, TG, TC, HDL-C, LDL-C were also determined. The level of insulin in the serum showed that insulin resistance in T2DM rat was ameliorated after PHF40-HD treated same as positive control group. There was a statistically significant (P<0.01) decrease in serum triglycerides (TG) below 2 mmol/L, but increase in serum high-density lipoprotein (HDL-C) exceed 1.4 mmol/L. Total cholesterol (CHOL) and low-density lipoprotein (LDL-C) versus model is not different. Treatment with Metformin for 30 days, positive control group rats compared to model control group rats, significantly decreased TG lever (P<0.01), yet the HDL-C lever remains unchanged in serum. PF40 did not have any effect on total cholesterol (TC) content in the serum (Table 3). The results indicated that PHF40 exhibited diverse effects on lipid metabolism.

**Table 3:** The detection results of insulin and lipid profiles in serum

**Groups**	**Doses**	**INS**	**CHOl**	**TG**	**HDL-C**	**LDL-C**
		**m U/L**	**mmol/L**	**mmol/L**	**mmol/L**	**mmol/L**
Model control		18.83 ± 3.66	1.97 ± 0.31	2.22 ± 0.72	1.29 ± 0.22	0.48 ± 0.06
Positive control		9.36 ± 2.43**	1.84 ± 0.32 ^C^	1.52 ± 0.27**↓	1.14 ± 0.29 ^C^	0.44 ± 0.05^C^
PF40	H	10.27 ± 2.70**	2.19 ± 0.19 ^C^	1.59 ± 0.23**↓	1.45 ± 0.15**↑	0.43 ± 0.05^C^
	M	18.04 ± 3.91c	2.17 ± 0.38 ^C^	1.98 ± 0.44**↓	1.40 ± 0.27**↑	0.42 ± 0.07^C^
	L	18.55 ± 2.37c	2.38 ± 0.11 ^C^	2.03 ± 0.43*↓	1.51 ± 0.07**↑	0.48 ± 0.03^C^

Values are expressed as mean ± S.d, n = 10.

Significantly different from the model group at* P ≤ 0.05, ** P ≤ 0.01.

^C^versus model is NOT different.

The sign “↓” expresses fall and “↑” expresses rise.

### OGTT and ITT

**Figure 2:**
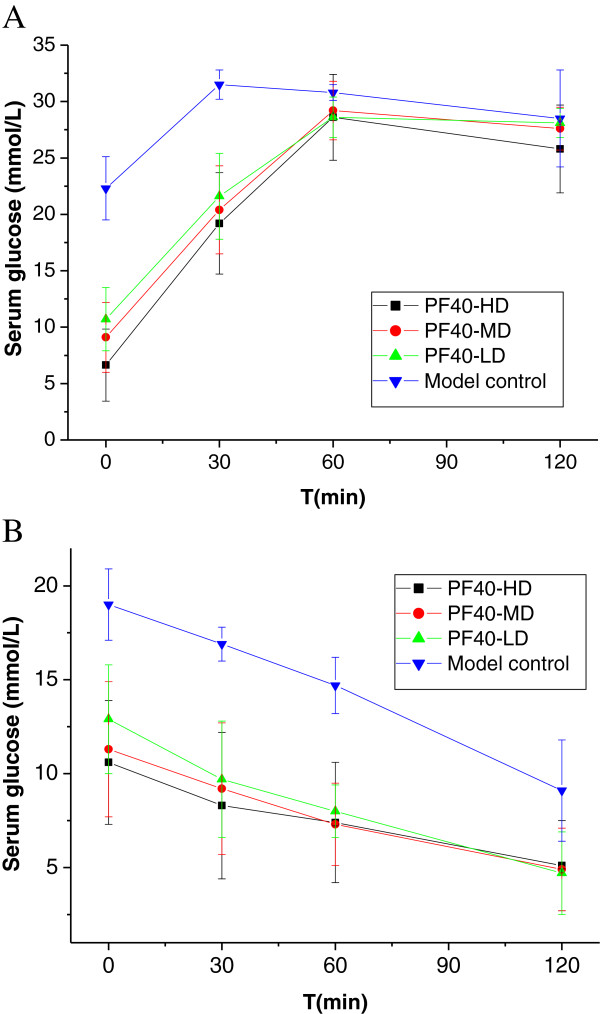
**The results of OGTT and ITT. A**. The result of Oral glucose tolerance test. **B**. The result of injecting insulin tolerance test.

After all the rats in groups were fed with glucose, serum samples were collected and detected at interval of 30 min up to 2 h. Blood glucose levels (mmol/L) in model rats elevated markedly in all time points with peaked at 30 min (31.5 ± 1.3), but all PF40 groups more gently rise in the blood glucose level than the model control group. Three PF40 treated groups suppressed the increment of blood glucose compared with model control group at 30 min (19.2 ± 4.5 *vs* 31.5 ± 1.3, 20.4 ± 3.9 *vs* 31.5 ± 1.3, 21.6 ± 3.8 *vs* 31.5 ± 1.3) that had significantly differences (p<0.01). It was regretted that PF40-HD, PF40-MD, and PF40-LD groups, blood glucose of diabetic rats moved up sharply with peaked at 60 min, the mean value of the glucose was no differences detected between model control group and treated groups. Either model group or PF40 groups, their blood glucose no returned close to base line (model group 22.3 ± 2.8 to 28.5 ± 4.3, PF40-HD group 6.64 ± 3.2 to 25.8 ± 3.9, PF40-MD group 9.1 ± 3.1 to 27.6 ± 1.9, PF40-LD group 10.7 ± 2.8 to 28.1 ± 1.3) and stop at a higher level than their initial at 120 min. Among PF40 groups, the PF40-HD did rats’ lower blood sugar a power of better. Where oral glucose tolerance test was concerned, the polysaccharide may not be very effective (Figure 2A).

After 30 days of treatment diabetic rats, the mean values (mmol/L) of a basal glucose were model group with 19.0 ± 1.9, PF40-HD group 9.6 ± 5.3, PF40-MD group 10.3 ± 3.6, PF40-LD group 11.9 ± 2.9, respectively. Injected insulin, the level of blood glucose continued to drop. In three PF40-treated groups were significantly lower than control at 30 min. Similarly, lower than control at 60 min (p<0.05). PF40 treatment improved glucose to regain a normal condition at 120 min. Among PF40s-treated groups, the higher dose-treated usually had more potent insulin tolerance than that of the lower dose and medial dose why it decreased blood-sugar steadily avoid abnormally low blood sugar usually resulting from excessive insulin (Figure 2B).

### Multiplex protein biomarker analysis results

**Figure 3:**
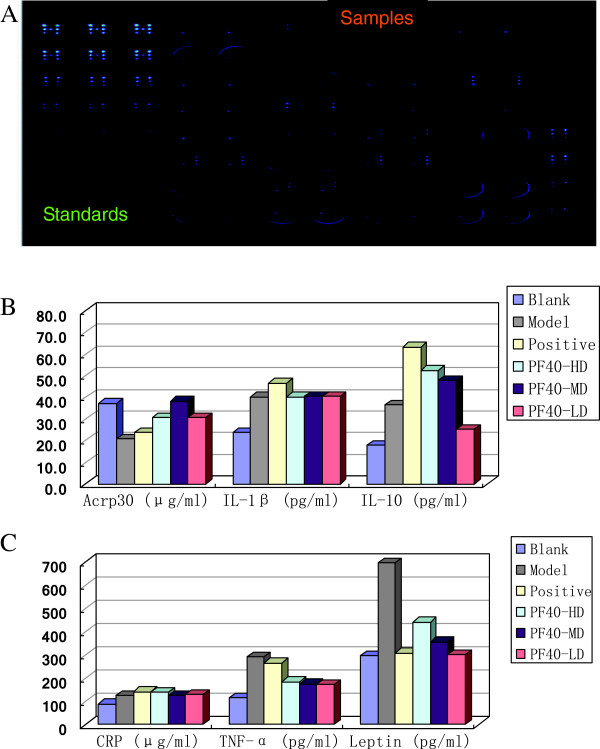
**The testing results of biomarkers of rats serum with multiplexing sandwich-ELISA method. A**. Serum was filled in 96-well plate collected CCD images of biomarkers from a ProArray Analysis System. Respective capture-antibodies are spotted in arrays within each well. 8 spots are for standard curve, each standard sample test in triplicates (left area). 6 group rat’s serum samples displayed the concentration profiles by quantitative chemiluminescence (right area). Samples test in duplicates, control test in duplicates for two different concentrations. **B**. The analysis results of 3 biomarkers Acrp30, IL-1β, and IL-10 in serum of T2DM rats after *Pseudostellaria heterophylla* polysaccharide (PF-40) treatment. **C**. The analysis results of 3 biomarkers CRP, TNF-α and leptin in serum of T2DM rats after *Pseudostellaria heterophylla* polysaccharide (PF-40) treatment.

SearchLight Protein Array Technology is a multiplexing sandwich-ELISA system based on chemiluminescent or fluorescent detection of analytes whose respective capture-antibodies are spotted in arrays within each well of a 96-well microplate. Measure 7 biomarkers per microplate well from 6 group rat’s serum samples displayed the concentration profiles by quantitative chemiluminescence. 8 spots for standard curve, each standard sample test in triplicates. Samples test in duplicates, control test in duplicates for two different concentrations. CCD imaging from each plate can be saved for a permanent record of raw experimental data, shown in Figure 3A. Type 2 diabetic model rats, the mean values of Acrp30 is lower than the blank control group in serum (20.87 ± 1.28 *vs* 37.25 ± 2.57 μg/ml); after treated with polysaccharide of PF40s, raise Acrp30 lever have basically recovered (38.22 ± 4.63, medial dose of PF40) which a medicinal effect get ahead of positive control. Type 2 diabetic model rats, raise the IL-1β in serum when compared with blank control group (40.25 ± 18.25 *vs* 23.84 ± 2.91, pg/ml); yet the target have no transformed to take PF40s. Innate immunoreactions was activated in type 2 diabetic model rats, anti-inflammatory cytokines IL-10 that have been increased in concentration in serum (36.58 ± 19.48 *vs.* 17.80 ± 3.94, blank control group); treated with metformin and PF40s got a positive increment suggested that they have obvious significant anti-inflammatory effects; the metformin and PF40-HD group, the levels of serum IL-10 were markedly increased reaching 63.36 ± 13.42 and 52.57 ± 17.49 pg/ml (p<0.01), see Figure 3B. Type 2 diabetic model rats, TNF-α is higher than the blank control group in serum (293.12 ± 41.67 *vs.* 114.10 ± 17.95, pg/ml), treated with PF40-LD got significantly differences with a negative increment 120.20 pg/ml (p<0.05), but curative effect of metformin is not good. Type 2 diabetic model rats, has a much higher leptin lever than blank control group (707.40 ± 106.07 *vs.* 298.24 ± 65.54); dropped to 303.16 ± 50.46 pg/ml had markedly differences after PF40-LD treatment (p<0.01), similar to metformin (310.00 ± 92.23 pg/ml). CRP is an acute phase reactant that increases markedly in response to inflammation, elevated serum CRP has been associated with an increased risk of type 2 diabetes. Rats took PF40s and fundamentally the CRP remains unchanged, that was no significantly differences compare with model control (Figure 3C). The concentration of IL-2 in serum was below quantification limit.

## Conclusions

Alloxan is one of the chemical agents that high lose of it used for the induction of type 1 diabetes mellitus in animal, alloxan has been found to be selectively toxic via GLUT2 into a pancreatic beta cell as it preferentially accumulates in theβ-cells as glucose analogues [22]. Type 1 diabetic mice were administered varied molecular weight polysaccharides from *Pseudostellaria heterophylla* on a diet for 21 days. The high does of PH-TP (from 7 to 210 kDa), the high and medial does of PF40 (from 50 to 210 kDa), and the high does of PF60 (from 10 to 210 kDa) lowered the levels of blood glucose as average were about 11.3 per cent lower than T1DM mice (p<0.05). It suggesting that they impossibly had the best effect to repair β-cell function, furthermore PF90 (from 3 to 6.8 kDa) was ineffective.

High-fat-diet/low lose of streptozotocin used for the induction of T2DM animal model characterized by abnormal metabolism of carbohydrates, fats and proteins, which is attributable to decreased insulin sensitivity leading to insulin resistance [2]. This paper has explored the PHPs effect on insulin resistance, adiponectin, leptin and inflammatory markers in type 2 diabetic rats.

Varied molecular weight polysaccharides from *Pseudostellaria heterophylla* oral administration to streptozotocin-induced T2DM rats, they were all able to significantly lower blood glucose. One of the four polysaccharides was the most effective, which was PF40 with molecular weight from 50 to 210 kDa. It could reverse the hyperglycemia and hyperlipemia status. ITT may be a useful method for assessing insulin resistance (IR) and predicting the effectiveness of insulin sensitizers [23]. The post-treatment in PF40 groups, in which insulin was given and blood glucose was measured at regular intervals. Thirty minutes after the insulin is administered, blood glucose level was lower but not less than half of the fasting glucose level. Glucose levels usually return to normal after about 90 minutes.

IL-10 is a pleiotropic cytokine inhibits the release of pro-inflammatory cytokines and counteracts the inflammatory process in order to re-establish homeostasis. Our experiments show that as IL-10 down-regulates the production of proinflammatory cytokines, the higher IL-10 levels were observed in obese type 2 diabetic rat models represent an attempt to inhibit continued proinflammatory cytokine production. The post-treatment in PF40 groups, the levels of the anti-inflammatory cytokine IL-10 were elevated continuously in T2DM rats. High TNF-α is related to the pathophysiology of IR and T2DM. TNF-α level was higher in rats with T2DM. The average level of TNF-α in PF40 groups were significantly lower than that in the model group. CRP is an acute-phase reactant produced primarily in the liver under the stimulation of adipocyte-derived IL-6 and TNF-α. A major mechanism by which CRP plays a critical role in T2DM is primarily by its action on pancreatic β-cell. Elevated glucose levels impair islet function and survival, and it has been proposed that intraislet expression of IL-1β contributes to glucotoxicity [24]. But it is a pity that the levels of CRP and IL-1β no changed after PF40 groups’ treatment.

Adiponectin (also known as Adipocyte complement-related protein of 30 kDa, Acrp30) is an adipocyte-derived protein with wide ranging paracrine and endocrine effects on metabolism and inflammation [25]. Insulin sensitivity is negatively correlated with obesity in type 2 diabetes, PF40 significantly increased the level of Acrp30 to promote adipocyte differentiation and fatty acid catabolism resulted that insulin was more sensitive. Leptin’s role in obesity prevents insulin resistance as novel therapeutic interventions of T2DM [26]. Although leptin in serum was increased in obesity T2DM rats, PF40 groups display reduced significantly it.

At present, in academic circles reach a consensus about the etiological factors of T2DM are complicated, and is hard to be treated. Our experiments show that *Pseudostellaria heterophylla* polysaccharide with the range of molecular weight is about 50 ~ 210 kDa (PF40) that could not only significantly lower blood sugar but also improve insulin tolerance. Increased the lever of anti-inflammatory cytokines IL-10 instead reduce inflammatory cytokines TNF-α to inhibit chronic low grade inflammation of the T2DM. Besides, PF40 could elevate the level of Acrp30 and reduce leptin and total triglyceride level in serum to regulate the disorder of fat metabolism of the T2DM. To compare with positive control metformin, adjusted the levels of Acrp30, IL-10, and TNF-α in serum, PF40 even had a much better effect. The content of leptin in serum had no difference between metformin with PF40. These demonstrated that PF40 can protect against the development of T2DM and improve IR via slow down overweight progress. We believe that polysaccharide of *Pseudostellaria heterophylla* is a promising anti-diabetic substance that will be helpful for the treatment of T2DM.

## Competing interests

The authors declare that they have no competing interests.

## Authors’ contributions

JH carried out the design of the study and coordinated the experiments and draft of the manuscript. WP carried out the extraction technology of polysaccharide and helped to draft the manuscript. JC purified polysaccharide, determined its molecule weight and composition. SB carried out the animal experiments. ZZ involved in the ELISA test. XW participated in the design of the study and performed the statistical analysis. All authors read and approved the final manuscript.

## Authors’ information

Juan Hu and Wensheng Pang tied for first place.

## Pre-publication history

The pre-publication history for this paper can be accessed here:

http://www.biomedcentral.com/1472-6882/13/267/prepub

## References

[B1] Holst JJ Vilsb¢II T Combining GLP-1 receptor agonists with insulin: therapeutic rationales and clinical findings Diabetes Obes Metab 2013 15 1 3 14 10.1111/j.1463-1326.2012.01628.x 22646532

[B2] Fang JG Wei HK Sun YY Zhang XD Liu WY Chang QT Wang RH Gong YW Regulation of podocalyxin expression in the kidney of streptozotocin-induced diabetic rats with Chinese herbs (Yishen capsule) BMC Complement Altern Med 2013 76 1 6 10.1186/1472-6882-13-76 PMC3637235 23560927

[B3] Li YJ Xu HX Research Progress on Anti · diabetic Chinese Medicines Zhong Yao Cai 2006 29 6 621 625 17039886

[B4] Grant SJ Chang DHT Liu JX Wong V Kiat H Bensoussan A Chinese herbal medicine for impaired glucose tolerance: a randomized placebo controlled trial BMC Complement Altern Med 2013 104 1 8 10.1186/1472-6882-13-104 PMC3659077 23672597

[B5] Teugwa CM Boudjeko T Tchinda BT Mejiato PC Zofou D Anti-hyperglycaemic globulins from selected Cucurbitaceae seeds used as antidiabetic medicinal plants in Africa BMC Complement Altern Med 2013 63 1 7 10.1186/1472-6882-13-63 PMC3618205 23506532

[B6] Guo XY Yoshitomi H Gao M Qin LL Duan Y Sun W Xu TH Xie PF Zhou JX Huang LS Liu TH Guava leaf extracts promote glucose metabolism in SHRSP.Z-Leprfa/Izm rats by improving insulin resistance in skeletal muscle BMC Complement Altern Med 2013 52 1 8 10.1186/1472-6882-13-52 PMC3599057 23452929

[B7] Pierre W Gildas AJH Ulrich MC Modeste W-N Benoît NT Albert K Hypoglycemic and hypolipidemic effects of Bersama engleriana leaves in nicotinamide/streptozotocin-induced type 2 diabetic rats BMC Complement Altern Med 2012 264 1 6 10.1186/1472-6882-12-264 PMC3546073 23267560

[B8] Manya K Champion B Dunning T The use of complementary and alternative medicine among people living with diabetes in Sydney BMC Complement Altern Med 2012 2 1 5 10.1186/1472-6882-12-2 PMC3295731 22240113

[B9] Jiang ZS Ni Q Zhou XZ Xing WJ Ling L Liu BY Study on drug law of Type 2 diabetes based on structured clinical information acquisition system J Shandong University of TCM 2007 31 3 159 203

[B10] Chen WN Jiang M Zheng G Guo HT Zhou Q Lv AP Exploring rules of Chinese patent medicines and Western medicines in treatment of type 2 diabetes with text mining Chinese J Exp Tradit Med Formulae 2011 17 21 299 302

[B11] Ni Q Chen SB Zhou XZ Wei ZX Gao YB Li Y Wang XM Li P Lin L Liu BY Study of relationship between formula (herbs) and syndrome about Type 2 diabetes mellitus affiliated metabolic syndrome based on free-scale networks Chinese Journal of Information on TCM 2006 13 11 19 22

[B12] Liu M Wu K Mao XQ Wu Y Ouyang JP Astragalus polysaccharide improves insulin sensitivity in KKAy mice: Regulation of PKB/GLUT4 signaling in skeletal muscle J Ethnopharmacol 2010 127 1 32 37 10.1016/j.jep.2009.09.055 19800959

[B13] Li WL Zheng HC Bukuru J De-Kimpe N Natural medicines used in the traditional Chinese medical system for therapy of diabetes mellitus J Ethnopharmacol 2004 92 1 1 21 10.1016/j.jep.2003.12.031 15099842

[B14] Chan CH Ngoh GC Yusoff R Brief review on anti diabetic plants: Global distribution, active ingredients, extraction techniques and acting mechanisms Pharmacogn Rev 2012 6 11 22 28 10.4103/0973-7847.95854 22654401 PMC3358964

[B15] Commission P Pharmacopoeia of the People’s Republic of China; Medical Science and Technology Press: Beijing China 2010 1 62 63

[B16] Pang WS Lin SD Dai QW Zhang HC Hu J Antitussive activity of pseudostellaria heterophylla (Miq.) pax extracts and improvement in lung function via adjustment of multi-cytokine levels Molecules 2011 16 4 3360 3370 21512444 10.3390/molecules16043360 PMC6260644

[B17] Xia LZ Xu XX Zhang R Efects of Pseudostellaria Polysaccharides on Glucose and Lipid Metabolism in Diabetic Rats China Pharmaceuticals 2009 l8 17 18

[B18] Ni SD Xia LZ Xu XX Zhang R Efect of pseudostellaria polysaccharides in diabetic mice by alloxan Anhui Medical and Pharmaceutical 2010 14 5 521 522

[B19] Xu JL Xu AR Ying JY Ma WC Chen YN Effect of Pseudostellaria Polysaccharides on Glucose and Insulin Metabolism on Diabetic Rats Chinese Arch Tradit Chinese Medicine 2012 30 2 423 424

[B20] Badawi A Klip A Haddad P Cole DEC Garcia BB El-Sohemy A Karmali M Type 2 diabetes mellitus and inflammation: Prospects for biomarkers of risk and nutritional intervention Diabetes, Metab Syndr Obes 2010 3 173 186 21437087 10.2147/dmsott.s9089 PMC3047967

[B21] Li YK Jin RM Wang QM Experimental Methodology of TCM Pharmacology 2006 Shanghai, China: Shanghai Science and Technology Press 603 607

[B22] Ankur R Shahjad A Alloxan induced diabetes: mechanisms and effects Int J Res Pharm Biomed Sci 2012 3 2 819 823

[B23] Duseja A Thumburu KK Das A Dhiman RK Chawla YK Bhadada S Bhansali A Insulin tolerance test is comparable to homeostasis model assessment for insulin resistance in patients with nonalcoholic fatty liver disease Indian J Gastroenterol 2007 26 4 170 173 17986744

[B24] Marianne BS Jeffrey T Géraldine P Lorella M Jan AE Julie KC Francois P Philippe AH Gordon CW Marc YD Increased Interleukin (IL)-1β Messenger Ribonucleic Acid Expression in β-Cells of Individuals with Type 2 Diabetes and Regulation of IL-1β in Human Islets by Glucose and Autostimulation Clin Endocrinol Metab 2008 93 10 4065 4074 10.1210/jc.2008-0396 PMC2579638 18664535

[B25] Yamauchi T Kamon J Waki H Terauchi Y Kubota N Hara K Mori Y Ide T Murakami K Tsuboyama-Kasaoka N Ezaki O Akanuma Y Gavrilova O Vinson C Reitman ML Kagechika H Shudo K Yoda M Nakano Y Tobe K Nagai R Kimura S Tomita M Froguel P Kadowaki T The fat-derived hormone adiponectin reverses insulin resistance associated with both lipoatrophy and obesity Nat Med 2001 7 941 946 10.1038/90984 11479627

[B26] Ravipati S Rajkuma B Role of Leptin in Diabetes Mellitus Indian J Fundam Appl Life Sci 2011 1 2 209 214

